# A Call to Digital Health Practitioners: New Guidelines Can Help Improve the Quality of Digital Health Evidence

**DOI:** 10.2196/mhealth.6640

**Published:** 2017-10-06

**Authors:** Smisha Agarwal, Amnesty E Lefevre, Alain B Labrique

**Affiliations:** ^1^ Bloomberg School of Public Health Johns Hopkins University Baltimore, MD United States; ^2^ Global mHealth Initiative Johns Hopkins University Baltimore, MD United States

**Keywords:** mHealth, checklist, reporting, digital health, publishing guidelines

## Abstract

**Background:**

Despite the rapid proliferation of health interventions that employ digital tools, the evidence on the effectiveness of such approaches remains insufficient and of variable quality. To address gaps in the comprehensiveness and quality of reporting on the effectiveness of digital programs, the mHealth Technical Evidence Review Group (mTERG), convened by the World Health Organization, proposed the mHealth Evidence Reporting and Assessment (mERA) checklist to address existing gaps in the comprehensiveness and quality of reporting on the effectiveness of digital health programs.

**Objective:**

We present an overview of the mERA checklist and encourage researchers working in the digital health space to use the mERA checklist for reporting their research.

**Methods:**

The development of the mERA checklist consisted of convening an expert group to recommend an appropriate approach, convening a global expert review panel for checklist development, and pilot-testing the checklist.

**Results:**

The mERA checklist consists of 16 core mHealth items that define what the mHealth intervention is (content), where it is being implemented (context), and how it was implemented (technical features). Additionally, a 29-item methodology checklist guides authors on reporting critical aspects of the research methodology employed in the study. We recommend that the core mERA checklist is used in conjunction with an appropriate study-design specific checklist.

**Conclusions:**

The mERA checklist aims to assist authors in reporting on digital health research, guide reviewers and policymakers in synthesizing evidence, and guide journal editors in assessing the completeness in reporting on digital health studies. An increase in transparent and rigorous reporting can help identify gaps in the conduct of research and understand the effects of digital health interventions as a field of inquiry.

## Introduction

Over the last decade, there has been a dramatic increase in health programs employing digital tools, such as mobile phones and tablets, to stimulate demand for or the delivery of health care services. This is especially true in low- and middle-income countries, where public health practitioners are tapping into the unprecedented growth in the use of mobile phones to overcome information and communications challenges [1,2]. Donors have rallied around digital approaches, and much has been invested into developing, testing, and deploying digital systems. However, after nearly a decade of concerted efforts, widely available evidence in support of digital health is limited [1,3,4]. As an emergent field, there is substantial variability in the reporting of digital program implementations, evaluations, and outcomes. Inconsistency in reporting is problematic as it limits policy makers’ ability to understand precise program details and extract, compare, and synthesize linkages (if any) between the digital investments and consequent health effects.

To address gaps in the comprehensiveness and quality of reporting on the effectiveness of digital programs, the mHealth Technical Evidence Review Group (mTERG)—an expert committee convened by the World Health Organization (WHO) to advise on approaches to strengthening digital health evidence—proposed guidelines for reporting evidence on the development and evaluation of digital health interventions. These guidelines—presented as the mHealth Evidence Reporting and Assessment (mERA) checklists—were published in March 2016 [5] and have since been widely accessed [1,6-10].

## Methods

The design of the mERA checklist followed a systematic process for the development of reporting guidelines [11]. In October 2012, WHO convened an expert working group led by the Johns Hopkins Global mHealth Initiative to develop an approach for the mERA guideline. In December 2012, this working group presented an initial draft of the checklist to a global panel of 18 experts convened by WHO during a 3-day meeting in Montreaux, Switzerland. At this meeting, the approach and checklist underwent intensive analysis for improvement, and a quality of information (QoI) taskforce was established to pilot-test the checklist. After testing by the QoI taskforce, the checklist and associated item descriptions were applied to 10 English language reports to test the applicability of each criterion to a range of existing mHealth literature. Readers may refer to further details about the methodology in the complete manuscript [5].

## Results

The mERA checklists comprises 2 components. The core mHealth checklist (see Table 1) identifies a minimum amount of information needed to define what the mHealth intervention is (content), where it is being implemented (context), and how it was implemented (technical features). This checklist may be valuable to researchers in reporting on the program and research results in peer-reviewed journals and reports, to policy makers in consolidating evidence and understanding the quality of information that has been used to generate the evidence, and to program implementers thinking through and selecting core elements for new digital health projects. L’Engle et al [12] applied the mERA checklist to evaluate the quality of evidence on the use of digital health approaches to improving sexual and reproductive health outcomes for adolescents. The study found that, on average, 7 out of 16 (41%) of the core mHealth checklist items were reported on, suggesting a lack of the availability of a clear description of the digital health intervention [12]. During the development and testing phase, the mERA checklist was applied to literature on the use of digital devices in reducing drug stockouts and the use of digital protocols to improve provider adherence to treatment protocols. Interested authors should refer to the definitions and examples for the core mHealth checklist available freely online [5].

**Table 1:** mHealth Evidence Reporting and Assessment (mERA) core checklist items.

Number	Item
1	Infrastructure
2	Technology platform
3	Interoperability/health information systems (HIS) context
4	Intervention delivery
5	Intervention content
6	Usability/content testing
7	User feedback
8	Access of individual participants
9	Cost assessment
10	Adoption inputs/program entry
11	Limitations for delivery at scale
12	Contextual adaptability
13	Replicability
14	Data security
15	Compliance with national guidelines or regulatory statutes
16	Fidelity of the intervention

**mHealth Evidence Reporting and Assessment (mERA) methodology.**

**Introduction**

1. Rationale/scientific rationale

2. Objectives/hypotheses

3. Logic model/theoretical framework

**Methods**

4. Study design

5. Outcomes

6. Data collection methods

7. Participant eligibility

8. Participant recruitment

9. Bias

10. Sampling

11. Setting and location

12. Comparator

13. Data sources

**Result**

14. Enrollment

15. Description of study population

16. Reporting on outcomes

**Discussion**

17. Summary of evidence

18. Limitations

19. Generalizability

20. Conclusions

**Conflicts**

21. Funding

22. Ethical considerations

23. Competing interests

**Additional criteria for quantitative study methods**

24. Confounding

25. Statistical methods

26. Missing data

**Additional criteria for qualitative study methods**

27. Analytic methods

28. Data validation

29. Reflexivity of account

The methodology checklist (see Textbox 1) outlines 29 items that highlight the key study design features that should be reported by researchers and evaluators of digital health interventions. Authors interested in using this checklist should note that there are other recommended checklists specific to different study designs—for example, Strengthening the Reporting of Observational Studies in Epidemiology (STROBE) for observational studies [13] and Consolidated Standards of Reporting Trials (CONSORT) for randomized trials [14]. We recommend that the core mHealth checklist be used in conjunction with these extant checklists based on the appropriate research study design that is being reported. However, we also recognize that a number of digital health studies that are being conducted to evaluate early-stage digital health interventions are more exploratory in nature, and the extant guidelines might not be as relevant to them. In such cases, the authors may decide to use the mERA methodology checklist, developed to be study-design agnostic, for reporting on the study design and results. A detailed explanation of the mERA methodology checklist items is available as a Web appendix [5].

## Discussion

We present an overview of the mERA checklist. For details about each of the checklist items under the core checklist items and the methodology items, we refer the readers to the complete publication [5]. The mERA checklist marks the culmination of several years of multiinstitutional collaborations, led by WHO, to determine appropriate standards for reporting on digital health evidence—standards that not only address issues of methodological and reporting rigor but also are responsive to the current state of the digital health space. We recognize that the digital health space is constantly evolving and is somewhat unique in its multidisciplinary nature, borrowing approaches from the fields of health care and technology and often engaging innovators who are unfamiliar with scientific methodologies. The mERA core and methodology checklists were pragmatically developed to be useful to a wide audience of innovators. We expect that the detailed explanations and examples make the checklist easy to use for individuals with varying levels of experience in academic reporting.

Even as the numbers of digital health interventions continue to increase, the evidence to support such interventions remains sparse. Without the support and shared commitment of the diverse digital health community in advancing the quality of evidence, the state of the much-critiqued “pilotitis” in mHealth will not change [15]. Transparency in the reporting of what constitutes a digital health intervention and clarity on evaluation methods are both critical to determining whether the digital strategy might be scalable to an entire population. In order to support the widespread adoption of the checklist, we encourage digital health researchers and program managers to ensure conformity with the checklist items. Additionally, we would like to call upon editors of journals publishing mHealth literature to encourage the use of the mERA checklist by presenting the link to the guidelines under Instructions to Authors and inclusion of a statement in the manuscript that “this manuscript was developed in conformity with the recommended criteria for reporting digital health as described in the mERA guidelines.”

## Abbreviations

CONSORT: Consolidated Standards of Reporting Trials
mERA: mHealth Evidence Reporting and Assessment
mTERG: mHealth Technical Evidence Review Group
QoI: quality of information
STROBE: Strengthening the Reporting of Observational Studies in Epidemiology
WHO: World Health Organization
